# Neuronal Nitric Oxide Synthase Regulates Depression-like Behaviors in Shortening-Induced Obese Mice

**DOI:** 10.3390/nu14204302

**Published:** 2022-10-14

**Authors:** Ping Wang, Fan-Zhi Kong, Xiao-Hong Hong, Li Zhang, Wan-Hong Zhao, Jin-Cui Yang, Heng Zhang

**Affiliations:** 1 Department of the Joint Laboratory of Biological Psychiatry, Mental Health Center of Shantou University, Shantou 515000, China; 2 Department of Medical Examination Center, Mental Health Center of Shantou University, Shantou 515000, China

**Keywords:** shortening, obesity, depression, neuronal nitric oxide synthase, 7-nitroindole

## Abstract

Shortening is mainly derived from the partial hydrogenation of palm oil and widely used in fast food. Food processed with shortening contains high levels of industrial trans fatty acids. Studies have shown that there is a correlation between industrial trans fatty acids, obesity, and depression. However, the regulatory effect of neuronal nitric oxide synthase (nNOS) on depression in obese patients is still unknown. The purpose of this study was to explore mood changes in obese mice fed a high shortening diet, and to determine the regulatory effect of nNOS on depressive-like behaviors in obese mice. We used a high shortening diet-induced obesity mouse model to systematically assess the metabolic response, behavioral changes, prefrontal and hippocampal nNOS protein levels, and the effect of nNOS inhibitors (7-nitroindole) on depression-like behavior in obese mice. Interestingly, obese mice on a 9-week high-shortening diet developed short-term spatial working memory impairment and anxiety-like behavior, and obesity may be a risk factor for cognitive impairment and mood disorders. In animals fed a high shortening diet for 12 weeks, obese mice developed depression-like behavior and had significantly elevated levels of nNOS protein expression in the hippocampus and prefrontal lobe. Administration of the nNOS inhibitor 7-nitroindole could improve depression-like behaviors in obese mice, further suggesting that inhibition of nNOS is helpful for depression associated with obesity.

## 1. Introduction

The advent of the fast food era has greatly contributed to the global prevalence of obesity [1]. Fast food products processed with industrially-produced, partially hydrogenated fat (margarines, shortening) are an important part of today’s fast food culture, providing enhanced flavor and prolonged storage [2,3]. Processed oils and fats contain high levels of industrial trans fatty acids, sometimes the reprocessed products reaching levels nearing 50% [4].Shortening is widely used in fried and baked foods. In a study on the distribution of trans fatty acids in modern foods in Germany, it was found that the trans fatty acid content index of shortening was ranked second [4]. Studies have shown that after industrial trans fatty intake, plasma levels of total cholesterol and low-density lipoproteins increase, whereas high-density lipoprotein levels decrease, and this increases the risk of obesity and metabolic diseases [5,6]. Epidemiological surveys have identified a bidirectional relationship between obesity and depression, with obesity increasing the risk of acquiring depression over time, and depression increasing the risk of obesity development in the future [7,8,9]. It has also been reported that there is an association between the chronic consumption of processed or fried foods and emotional disorders, including anxiety and depression [10]. Similarly, trans fatty acid consumption has been reported to increase the risk of depression [11]. These studies highlight the correlation between industrially-produced, partially hydrogenated fat, obesity, and depression.

Animal experiments have demonstrated that after long-term feeding of a highly saturated fat diet, obese mice developed systemic inflammation, impaired spatial memory [12], and anxiety and depression-like behaviors [13,14,15]. Anxiety and depression can increase food cravings that can lead to a deleterious cycle that can increase the risk of obesity and emotional disorder comorbidities [16,17]. Previous studies have relied on the use of saturated fatty acids to establish animal models of obesity. Industrially-produced, partially hydrogenated products contain large amounts of industrial trans fatty acids that are different from saturated fatty acids in sources, structures, and proinflammatory abilities [18]. Multiple biological pathways are involved in the depression-obesity link, including genetics, HPA axis abnormalities, immuno-inflammatory activation, alterations in neuroendocrine regulators of energy metabolism and brain circuitries integrating mood regulatory responses [19]. However, it is unknown whether cognitive and mood changes occur in industrially-produced, partially hydrogenated fat (shortening)-induced obesity mouse models.

Nitric oxide synthase (NOS) is a protein that catalyzes the production of nitric oxide (NO) from L-arginine. NOS has three isoforms: neuronal NOS (nNOS) (the isoform identified first found predominantly in nerve tissue), inducible NOS (iNOS), and endothelial NOS (eNOS). In the central nervous system, NO is both a neurotransmitter and a neuromodulator, playing an important role in synaptic and intercellular information transmission [20]. Clinical studies have shown that there is increased nNOS expression in the hippocampal (CA1) and hypothalamic regions in patients with bipolar disorder [21]. Serum NO levels in patients with depression increase significantly [22], while partial exhaled NO (an indicator for waste-handling capacity) levels in patients with depression decrease [23]. Animal experiments have demonstrated that the chronic mild stress (CMS) induced depression model could selectively upregulate the expression of nNOS in the hippocampus, and inhibiting nNOS could prevent and reverse CMS-induced depression [24]. Inhibition of nNOS can also improve spatial memory impairment induced by lipopolysaccharide (LPS) in rats [25]. However, there are inconsistent reports suggesting that nNOS activity in the prefrontal cortex of patients with major depressive disorder is significantly reduced [26]. Strikingly, the depression-like behavior of ovariectomized mice could be alleviated by increasing nNOS levels in the hippocampus, with nNOS inhibition capable of reversing antidepressant-like effects [27]. However, the mixed results observed in the above studies clearly show that the relationship between nNOS and depression is still unclear. It has been previously reported that long-term saturated fat diets increase the expression of nNOS protein in the hippocampus and cerebral cortex [28]. Whether obesity induced by shortening can increase the expression of nNOS protein in related brain regions and regulate depression-like behavior in obesity models requires further exploration.

The goal of this research was to explore mood changes in obese mice fed a shortening diet, and to determine the regulatory effects of nNOS on depressive-like behavior during obesity. We performed systematic metabolic and behavioral assessments of obese mice models (high shortening diet; ~12 weeks), measured changes in nNOS protein levels in the hippocampus and prefrontal cortex, and investigated the effect of 7-nitroindole (nNOS inhibitor) on depression-like behavior in obese mice.

## 2. Materials and Methods

### 2.1. Animals

All animal procedures were performed in accordance with the guidelines of the National Institute of Health for guiding animal research and approved by the Laboratory Animal Management and Use Committee of Shantou University Medical College (Shantou, China; SUMC2020-055). Seven-week-old male C57BL/6N mice (*n* = 44) were purchased from Beijing Vital River Laboratory Animal Technology Co., Ltd. (Beijing, China). Three animals/cage were housed together in rectangular polypropylene cages with constant temperature (22–24 °C), humidity (33–35%) and light-dark cycle (12–12 h) controls. Animals were allowed to drink water and food at will. After 1 week of adaptive feeding, mice were randomly assigned to one of two customized diets (Table 1; dietary composition, Beijing HFK Bioscience Co., Ltd., Beijing, China): (1) Control diet (CD): a 16.8% kcal soybean oil diet (*n* = 19, AIN-93G); and (2) High shortening diet (HSD): a 50% kcal shortening (partially hydrogenated palm oil) diet (*n* = 25, Modified AIN-93G).

### 2.2. Study Design

**Figure 1:**
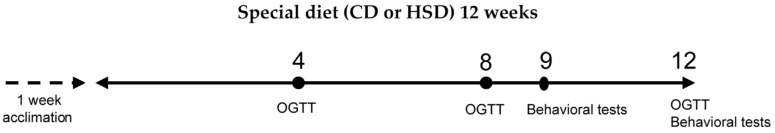
Experimental flow chart.

Body weights and fasting blood glucose measurements were performed weekly, and oral glucose tolerance was tested at weeks 4, 8, and 12 post initiation of custom diet feeding. Diet-induced obesity (DIO) mice were defined as those in HSD group weighed more than the heaviest mouse in the control group [13]. HSD mice weighing less than the heaviest control were excluded from the behavioral test. We conducted behavioral experiments at weeks 9 and 12, respectively. At the beginning of the experiment, there were 19 mice in CD and 25 mice in HSD. At week 9, we obtained 18 DIO mice from HSD group. Additionally, the Y-maze (Day 1), elevated plus maze (Day 2), and open field test (Day 3) were performed in DIO mice (*n* = 18). By continuing HSD feeding, the number of DIO mice increased to 20 at week 12. The sucrose preference test was performed in DIO mice (*n* = 20). Western blot analysis for nNOS was performed in the prefrontal lobe and hippocampus from five CD to six DIO mice. Blood samples were obtained retro-orbitally. The remaining mice of CD (*n* = 14) and DIO (*n* = 14) were divided into four groups (seven mice in each group): CD group, DIO group, CD + 7-NI group (7-NI, 7-nitroindole, a selective inhibitor of nNOS) and DIO + 7-NI group. After intraperitoneal injection (i.p.) of 7-NI (Sigma, St. Louis, MO, USA; 30 mg/kg) or vehicle control [24] for 2 h, the open field test and forced swimming test were performed. After the behavioral experiments, blood samples were collected, and tissues were harvested for downstream analysis. Experimental flow chart (Figure 1).

### 2.3. Fasting Blood Glucose Test and Oral Glucose Tolerance Test (OGTT)

After 12 h of fasting, mouse body weights and fasting blood glucose (One Touch) measurements were tested at 8 am the following day. OGTT was performed at different times (4, 8, and 12 weeks of HSD or CD). After an overnight fasting period, baseline blood glucose values were obtained using a glucometer, followed by gastric perfusion with a 20% glucose (2 g/kg, i.g.) solution. Blood glucose levels were measured at 30, 60, 90, and 120 min after gastric perfusion.

### 2.4. Behavioral Tests

#### 2.4.1. Y-Maze

For the Y-maze test, mice were placed in the center of a Y-shaped device composed of three identical closed arms (arm length: 40 cm, arm bottom width: 3 cm, arm upper width: 13 cm, height of wall: 15 cm). Each mouse was placed in the central area. The short-term work memory of mice was assessed by measuring the number of all arm entries, the correct number of arm entries, and the spontaneous alternation percentage accomplished within 5 min. The correct number of arm entries was defined as consecutive entries into all three arms. For example, entering the ABCABC arm region, the correct number entering the arm is four, namely ABC, BCA, CAB, ABC.

#### 2.4.2. Open Field Test

For the open field test (OFT), mice were placed in in the center of a square box (50 × 50 × 50 cm) to evaluate their movement distance within 5 min, along with the time and distance spent in the central area, which is indicative of the exercise capacity and anxiety-like behaviors of the mice. The center zone was defined as a square, 12.5 cm away from the wall.

#### 2.4.3. Elevated Plus Maze

The elevated plus maze (EPM) consisted of two open arms and two closed arms (40 cm length, 10 cm width, 50 cm height), always facing the open arms. Mice were placed in the central area of the maze facing one of the open arms. Anxiety-like behavior was assessed by quantifying the number and time of entering the open arms within 5 min.

#### 2.4.4. Sucrose Preference Test (SPT)

Mice were exposed to a 1% sucrose solution for 48 h, with the position of sucrose and water exchanged every 24 h. On the third day, mice were exposed to two identical bottles. One bottle was filled with 1% sucrose, and the other bottle was filled with ordinary water. Sucrose preference was defined as the ratio of sucrose consumption volume to total liquid consumption volume within 24 h, which is an assessment of anhedonic behavior (one of the core symptoms of depression).

#### 2.4.5. Forced Swimming Test (FST)

Mice were forced to swim in a glass cylinder (height, 15 cm; diameter, 12 cm) containing water (23 °C) at a 10-cm depth for 6 min. Habituation was determined in the first 2 min of the experiment, and the time of immobility in the last 4 min was indicative of behavioral despair.

### 2.5. Blood Lipid Measurements

Blood was left to coagulate at room temperature (26 °C, 2 h). Supernatants collected after centrifugation were used to measure total cholesterol, triglycerides, and low- and high-density lipoprotein levels.

### 2.6. Western Blotting

Brain tissue was homogenized in ice-cold protein lysis buffer (CST, USA) and centrifuged to obtain cytoplasmic proteins. The protein concentration was determined by bicinchoninic acid (BCA) protein assay. Proteins from each sample (10 µg total protein per lane) were separated by SDS-PAGE constant pressure electrophoresis, transferred to polyvinylidene fluoride (PVDF) membranes under constant pressure, blocked in 5% skim milk at 4 °C overnight, and incubated with primary antibodies against nNOS (1:1000, CST, USA) and β-actin (1:1000, CST, USA) at 4 °C overnight. The following day, membranes were washed three times with phosphate buffered saline (PBS) and incubated with horseradish peroxidase-conjugated anti-rabbit IgG secondary antibody (1:200, Beyotime, China) for 2 h. Chemiluminescence was detected using ECL color solution and analyzed with image analysis software (Image J).

### 2.7. Statistical Analysis

All experiments were conducted using a completely randomized design, with all behavioral experimental data analyzed using the animal behavior analysis system (EthoVisionXT). If the data conformed to the normal distribution, they were expressed as the mean ± standard deviation (SD); otherwise, they were expressed as the median (quartile spacing). A *t*-test was performed to compare two groups when the data conformed to the normal distribution, otherwise a Mann–Whitney *U* test was performed. If the data conformed to the normal distribution, correlations were assessed by Pearson correlation analyses, otherwise a Spearman correlation analysis was assessed. A two-way ANOVA was performed to analyze mouse body weights, blood glucose measurements, and inhibitor 7-NI intervention studies. The level of significance was set as *p* < 0.05.

## 3. Results

### 3.1. HSD Exposure Increases Bodyweight and Leads to Abnormal Glucose and Lipid Metabolism

**Figure 2:**
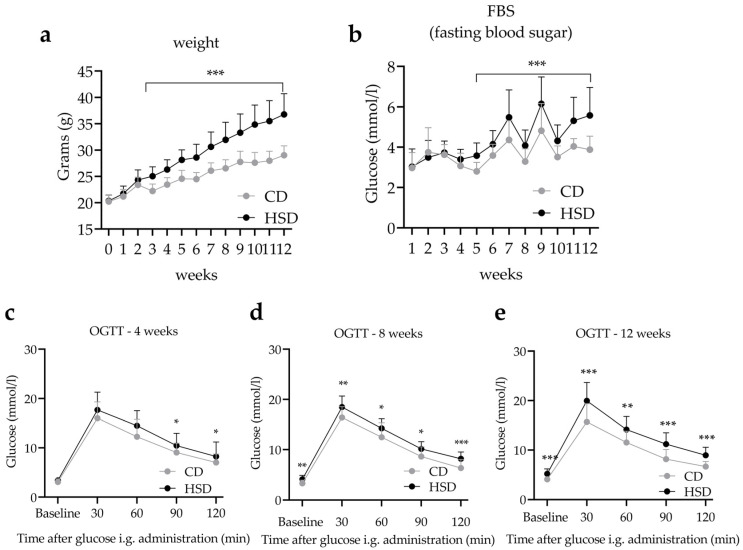
HSD exposure leads to bodyweight gain, glucose tolerance impairment, and hyperglycemia. (**a**) Bodyweight from 0–12 weeks of mice fed CD or HSD. (**b**) Fasting blood glucose of CD or HSD mice fed for 1–12 weeks. (**c**–**e**) Oral Glucose Tolerance Test (OGTT) of CD or HSD fed mice at weeks 4, 8, and 12. * *p* < 0.05, ** *p* < 0.01, *** *p* < 0.001. When compared with CD group, results are presented as the mean ± SD.

Before diet exposure, equal weights and similar average body weights were observed in both groups (*t* = 0.35, *df* = 42, *p* = 0.72). We observed significant increases in body weight after 12 weeks of HSD feeding [*F* (1,42) = 56.84, *p* < 0.001] with a distinct interaction identified between time and diet [*F* (1,42) = 6.40, *p* < 0.001], indicating that the body weights of animals in the HSD group were significantly higher than the CD group (Figure 2a). Three weeks of HSD were required to induce significant weight gain between the two groups, with the HSD mice gaining 16.7% compared to the controls (*t* = 5.81, *df* = 42, *p* < 0.001). Weights of the animals in the HSD group remained higher than controls throughout the entirety of the experiment, with a gain of 26.7% compared to the controls after 12 weeks (*t* = 7.92, *df* = 42, *p* < 0.001). Five weeks of HSD were required for fasting blood glucose impairment in the HSD group, which persisted during the remainder of the experiment (Figure 2b). Next, animals were subjected to an OGTT, a diagnostic tool for diabetes that reflects the body’s metabolic efficiency and insulin resistance [29]. Blood glucose levels increased after glucose administration and dropped 60 min after glucose administration in both groups. After 90 min, we observed significant differences in blood glucose levels between the two groups, which lasted until the 120-min timepoint (Figure 2c). These data indicate that 4 weeks of HSD exposure significantly decreased glucose clearance, leading to aberrant glucose metabolism during later timepoints post HSD diet implementation (Figure 2d,e). We also observed a significant relationship between time and diet after 12 weeks of HSD compared to the CD group [*F* (1,42) = 3.67, *p* < 0.05], suggesting that the rate of glucose metabolism in HSD fed animals was significantly lower.

**Figure 3:**
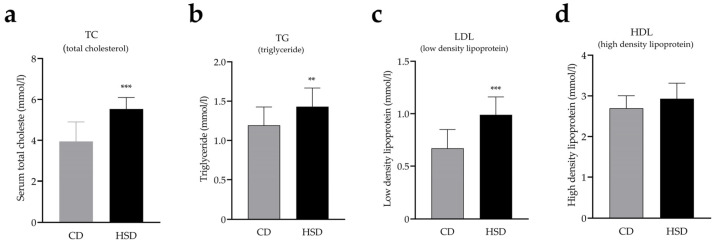
HSD mice have disordered lipid metabolism. ** *p* < 0.01, *** *p* < 0.001. When compared with CD group results are presented as the mean ± SD.

At week 12, the serum total cholesterol (TC, Figure 3a, *t* = 5.91, *df* = 32, *p* < 0.001), triglyceride (TG, Figure 3b, *t* = 2.93, *df* = 32, *p* < 0.01), and low-density lipoprotein (LDL, Figure 3c, *t* = 5.42, *df* = 32, *p* < 0.001) levels of the HSD group were significantly higher than the control, but high-density lipoprotein levels were not statistically different (HLD, Figure 3d, *t* = 1.97, *p* = 0.06). These results indicate that mice fed an HSD for 12 weeks had glucose and lipid metabolism disorders.

**Figure 4:**
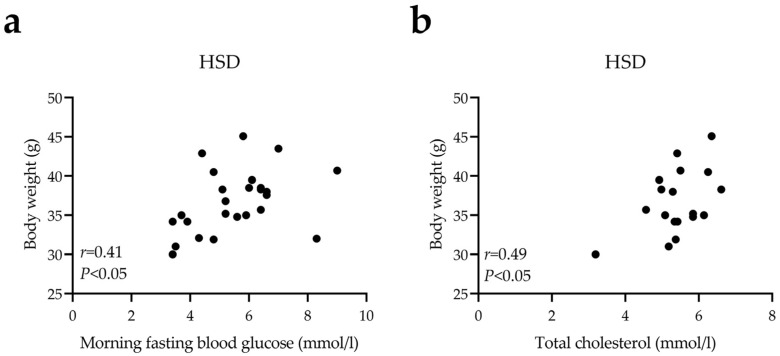
HSD-induced body weight was associated with morning fasting blood glucose (**a**) or total cholesterol (**b**).

After 12 weeks, we identified a positive correlation between body weight and morning fasting blood glucose (Figure 4a, *r* = 0.41, *p* < 0.05) or TC levels (Figure 4b, *r* = 0.49, *p* < 0.05) in the HSD group, further implicating obesity as a risk factor for hyperglycemia and hyperlipidemia.

### 3.2. Short-Term Spatial Working Memory Impairment, Anxiety-like, and Depression-like Behaviors in DIO Mice

**Figure 5:**
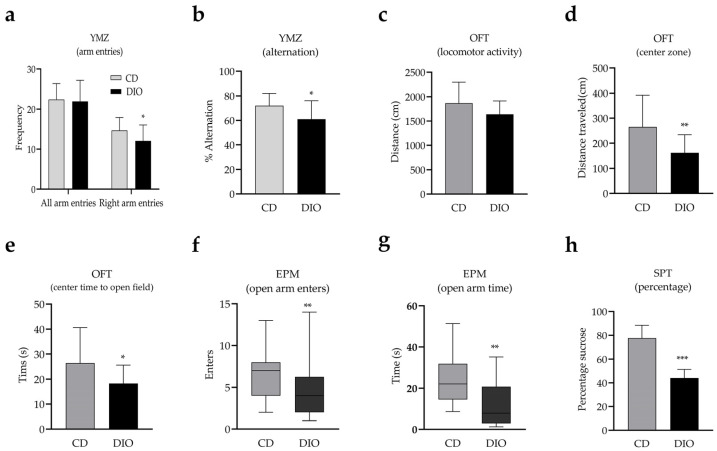
Diet-induced obesity (DIO) mice show impaired short-term spatial working memory, anxiety-like, and anhedonic behaviors. Arm entries (**a**) and alteration (**b**) in elevated plus maze (YMZ), locomotor activity (**c**) in open field test (OFT), center distance traveled (**d**) and time (**e**) in OFT, number of entries (**f**) and time spent (**g**) in open arms of elevated plus maze (EMP), sucrose preference percentage (SPT) (**h**). * *p* < 0.05, ** *p* < 0.01, *** *p* < 0.001. When compared with the CD group, results were presented as the mean ± SD or median (quartile spacing).

In order to explore the short-term memory and mood changes in obese mice, behavioral experiments were performed in DIO mice. The Y-maze test was performed to evaluate the short-term spatial working memory. There was no statistical difference in the total number of arm entries between the CD and DIO group (Figure 5a, *t* = 0.29, *df* = 35, *p* = 0.78). However, there were significant differences in the number of right arm entries (Figure 5a, *t* = 2.16, *df* = 35, *p* < 0.05) and the spontaneous alteration percentage (Figure 5b, *t* = 2.74, *df* = 35, *p* < 0.05) between the CD and DIO group. These results suggest that animals in the DIO group experienced an impairment in short-term spatial working memory, but not in spatial exploration ability compared to CD mice. The open field test (OFT) was performed to measure locomotive activity and anxiety-like behaviors between the two groups. The total distance traveled in 5 min was comparable between both groups (Figure 5c, *t* = 1.91, *df* = 35, *p* = 0.06). In contrast, both the distance traveled (Figure 5d, *t* = 2.97, *df* = 35, *p* < 0.05) and time spent (Figure 5e, *t* = 2.09, *p* < 0.05) in the center zone were significantly lower in the DIO group than CD group. In the elevated plus maze test (EMP), DIO animals had significantly lower measurements for the number of times entering the open arms (Figure 5f, *df* = 35, *p* < 0.05) and time spent in the open arms (Figure 5g, *df* = 35, *p* < 0.05) compared to control animals, indicating anxiety-like behavior. The sucrose preference test (SPT) is a measurement of anhedonic behavior as a core symptom of depression [30]. In this study, we observed a significant decrease in the preference for sucrose solution by DIO mice compared with the CD group (Figure 5h, *t* = 10.15, *df* = 29, *p* < 0.001). In summary, the behavioral studies performed demonstrate that DIO mice develop short-term spatial working memory impairment, anxiety-like, and anhedonic behaviors in response to HSD feeding.

**Figure 6:**
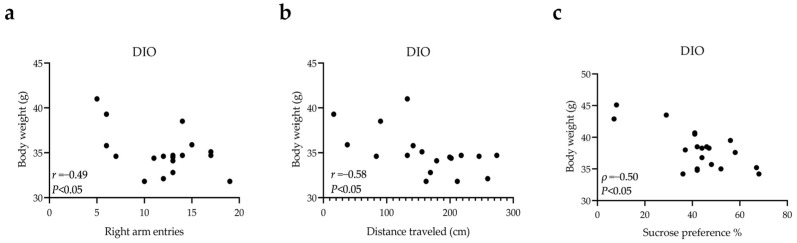
The body weight of DIO mice was negatively correlated with the right arm entries (**a**) or distance traveled in the central zone (**b**) or sucrose preference percentage (**c**).

Next, the relationship between body weight and behavioral traits was further explored in the DIO group. At week 9, we found that body weight was negatively correlated with the number of correct entries during the YMZ test (Figure 6a, *r* = −0.49, *p* < 0.05) or distance traveled in the central zone in during the OFT test (Figure 6b, *r* = −0.58, *p* < 0.05). Furthermore, we found a negative correlation between body weight and sucrose preference percentage at week 12 of HSD feeding (Figure 6c, *ρ* = −0.50, *p* < 0.05). Ultimately, these results suggest that obesity may be a risk factor for anxiety- and depression-like behaviors.

### 3.3. Changes of nNOS Protein Levels in the Brain of DIO Mice

**Figure 7:**
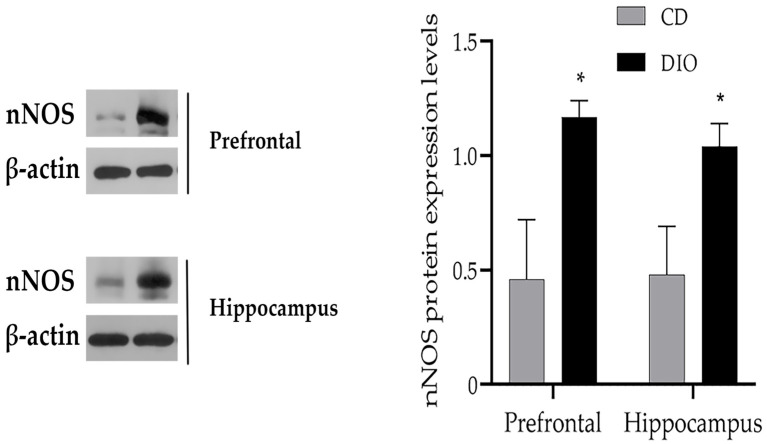
The expression of nNOS in the prefrontal cortex and hippocampus of DIO mice increases significantly. * *p* < 0.05. When compared with the CD group, the results were presented as the mean ± SD.

Compared with CD group, nNOS protein levels in both the hippocampus (*t* = −4.09, *df* = 9, *p* < 0.05) and prefrontal cortex (*t* = −4.57, *df* = 9, *p* < 0.05) increased significantly in the DIO group (Figure 7).

### 3.4. Depression-like Behavior Improved by 7-NI in DIO Mice

**Figure 8:**
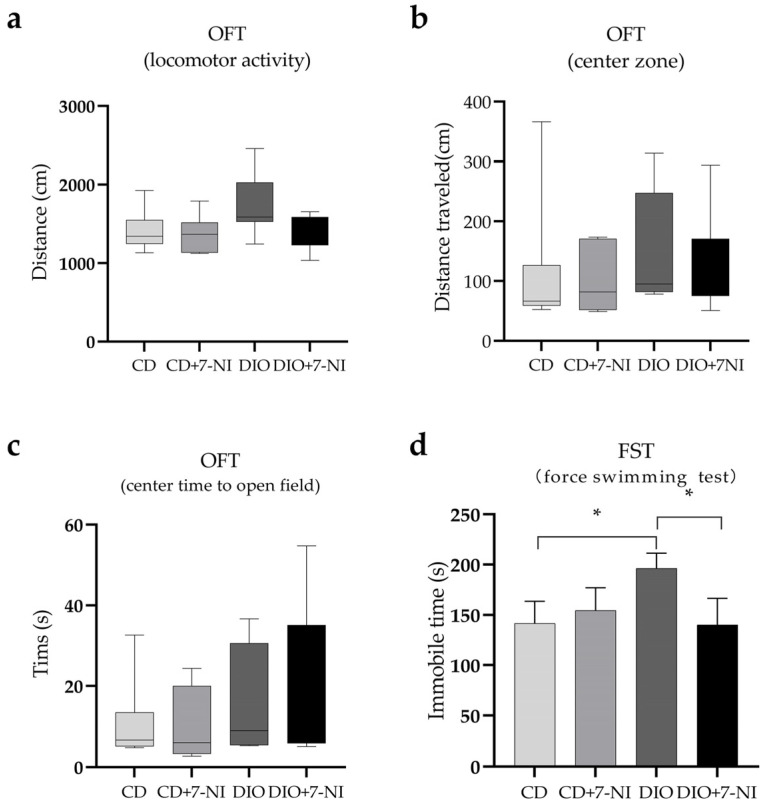
7-NI improves depressive behavior in DIO mice. Exercise ability (**a**), central travel distance (**b**) and time spent (**c**) by DIO mice in during OFT. The immobility time of mice in each group during forced swimming (**d**). * *p* < 0.05, When compared with the corresponding group, results are presented as the mean ± SD or median (quartile spacing).

Due to the influence of 7-NI on altering the motor ability of mice [31], the OFT was performed in mice after injection of 7-NI or vehicle control for 2 h. The results of OFT were not significantly different in the total movement distance, central area movement distance, or time spent amongst the four groups (*p* > 0.05) (Figure 8a–c). Furthermore, we observed a significant difference in the immobility time during FST between DIO and CD mice (*p* < 0.05), with no significant difference observed in immobility time between the CD and CD + 7-NI groups (*p* < 0.05). Interestingly, the immobility time in the DIO + 7-NI group was reduced by 29% compared with the untreated DIO group (*p* < 0.05). In short, these data indicate that 7-NI may ameliorate the depressive-like behaviors in DIO mice (Figure 8d).

## 4. Discussion

In this research, we found that HSD-fed obese mice developed cognition impairment, anxiety and depression-like behaviors. Compared with the control group, nNOS protein levels in the prefrontal cortex and hippocampal regions were significantly greater in obese mice. Interestingly, the depressive-like behaviors of obese mice associated with HSD improved after treatment of obese mice with 7-NI.

Animal models of obesity are important to investigate mechanisms of obesity and related comorbidities in preclinical status, although there are differences in physiological studies between obese animal models and human obesity [32]. Compared with genetic models, human obesity reproduced by diet-induced obesity animal models is more reliable [32]. Diet-induced obesity leads to progressive weight gain and insulin resistance in mice, similarly to the pathogenesis of obesity in human [33]. In our study, the changes we observed in the HSD group were consistent with the pathogenesis of obesity in terms of body weight, fasting blood glucose, and glucose tolerance. As obesity models, C57BL/6J mice are prone to show characteristics of obesity when they are fed a high-fat diet [34]. For C57BL/6J mice younger than 8 weeks of age, high-fat diets are not significantly associated with subsequent obesity [35]. Additionally, male C57 mice are more prone to obesity than female C57 [36]. C57BL/6J mice have a nicotinamide nucleotide transhydrogenase gene mutation that inhibits insulin secretion and mitochondrial function [37]. Therefore, male C57BL/6N mice of 8 weeks were chosen for our study. The World Health Organization defines obesity as abnormal or excessive fat accumulation that may impair health. Based on the purpose of our experiment, obese mice needed to be evaluated at a different stage. There is no standardized definition of obesity that can presently be applied to animal models [33]. Considering experimental design and ethics, we chose to define diet-induced obesity animal model by weight. Compared with highly saturated fatty acids, HSD induces weight gain much earlier [14]. In line with this, we found that mice fed an HSD rapidly gained weight, and developed glucose and lipid metabolism disorders, suggesting that obesity is a risk factor for hyperglycemia and hyperlipidemia.

In the behavioral experiments, there were no significant differences in the total number of entries or total movement distance of the mice in each group, suggesting that body weight did not affect exploration or exercise abilities. Furthermore, we observed an induction of short-term spatial working memory impairment and anxiety and depression-like behaviors in HSD obese mice, which is consistent with previous studies [12,13,14,15]. However, some studies have shown that high fat diet does not cause anxiety and depression-like behaviors in mice [38], and can have reinforcement cognitive function [39], antianxiety, and antidepressant effects [40]. The possible reasons for these discrepancies include small sample sizes [38], shorter feeding periods [39], variability in strain and age of mice, and varied diet compositions [40]. We also analyzed the correlation between body weight and behavior in obese mice and found that HSD-induced obesity was associated with cognitive impairment and mood disorders.

Previous animal studies have shown that a long-term high-fat diet could increase markers of inflammation (such as interleukin-6 (IL-6), interleukin-1β (IL-1β), and tumor necrosis factor-α (TNF-α)) in the hippocampus and nucleus accumbens [12,13,14], impair the dentate gyrus (DG) of the hippocampus and 5-hydroxytryptamine signaling [41], and affect the plasticity of dopamine in the nucleus accumbens and amygdala [42]. Abnormal transmission of monoamines and damage to brain neurons leads to cognitive impairment and emotional disturbance [19]. It could be speculated that mental disorders induced by chronic high-fat diets may involve abnormal inflammation and signal transduction in multiple brain regions, especially in the hippocampus. It is believed that the pathophysiology of depression involves abnormalities in the prefrontal cortex and hippocampus [43].

In this study, we found that the expression level of nNOS protein increased significantly in the hippocampus (consistent with the study of saturated fat-fed mice [28]) and prefrontal lobe of mice in the DIO group. Nitric oxide synthase degradation is regulated by the ubiquitin proteasome pathway [44], while high-fat and high-sugar diets alter protein ubiquitination [45]. This could shed light on the reason for the significant increase in nNOS protein expression levels in DIO mice rather than CD mice. High levels of nNOS protein may lead to the excessive production of NO, excessive NO signals through nitrification, nitrosation, and oxidative modification into reactive nitrogen species, which are usually toxic to cells [43]. This suggests that impairment of cognitive function and mood disorders may be associated with high levels of nNOS in DIO mice.

7-NI is a fast and reversible nNOS inhibitor, which competitively binds to the tetrahydrobiopterin (nNOS cofactor) sight, leading to inactivation of the nNOS dimer and inhibition of NO production [46]. However, inactivity of the nNOS dimer is not a signal of protein degradation because 7-NI may stabilize proteins [47]. The monomeric form of NOS may have more sights for ubiquitination, allowing it be hydrolyzed more easily by trypsin [48]. In summary, 7-NI can delay the metabolism of nNOS in the short term. Therefore, the regulation of nNOS protein levels by 7-NI in the short term was not determined in this study. The forced swim test (FST) is an assay for the study of depressive-like behavior in mice [49,50], and the duration time of immobility reflects the degree of behavioral helplessness [51]. In FST studies, we found that the immobility time of DIO mice was reduced significantly when nNOS was inhibited by 7-NI treatment, suggesting that nNOS is involved in the pathophysiology of depressive-like behavior in HSD fed mice, which could be ameliorated by 7-NI. It has been reported that when 7-NI is administered alone into the raphe nucleus, the release of serotonin increases in the frontal cortex [52]. Moreover, local perfusion (into the ventral hippocampus, 1 mM) and systemic (50 mg/kg and 30 mg/kg) administration of 7-NI increases the outflow of serotonin by the ventral hippocampus in rats [53]. Therefore, the improvement of mood disorders in the DIO group by 7-NI in this study may also be related to the release of serotonin; however, further studies are needed to confirm this idea.

Numerous studies have shown that NO is a central regulator of energy metabolism in obese animal models and obese patients [54]. In a mouse obesity model, iNOS is strongly activated in hypothalamic arcuate nucleus macrophages, and inhibition of iNOS can eliminate central inflammation and improve glucose metabolism [55]. The sensitivity to satiety signals increases and energy intake is reduced after mice fed a high-fat diet are treated with an iNOS inhibitor [56]. In our study, we found that nNOS had a regulatory effect on the depressive-like behavior observed in obese mice, further demonstrating an important role of the nitrogenergic nervous system in obesity-related mood and metabolic disorders.

## 5. Conclusions

We found obese mice induced with a long-term HSD led to the development of cognitive impairment and mood disorders. Inhibition of nNOS could improve the depression-like behavior of obese mice, which provides a new perspective for the treatment of depressive emotions associated with obesity. However, there were several important limitations of this work. First, the inflammation variations in obesity were not explored. Second, the restriction of the use of male mice in the work was also an important limitation. Third, the failure to assess body fat by direct chemical measurement was also a weakness. Quantification of the body fat content of an individual mouse would be more precise and accurate, hence providing sufficient degrees of freedom for a meaningful statistical analysis. At present, most countries do not enforce a ban on the production of industrial trans fats [57]. The increasing prevalence of obesity is associated with the prevalence of industrial processed trans fatty foods. Moreover, the increased risk of depression is common in obese individuals. Our findings highlight an urgent need to continue the study of the pathological basis of depression in obese patients to find better treatment options for affected individuals.

## Figures and Tables

**Table 1:** Diet composition.

	CD	HSD
Fat source	Soybean oil	Partially hydrogenated palm oil
Fat (g/kg)	70	270
Casein (g/kg)	200	200
L-Cystine (g/kg)	3	3
Sucrose (g/kg)	100	100
Cornstarch (g/kg)	397.5	197.5
Dyetrose (g/kg)	132	132
Mineral Mix (g/kg)	35	35
Vitamine Mix (g/kg)	10	10
Choline Bitartrate	2.5	2.5
Cellulose	50	50

Control diet (CD); High shortening diet (HSD).

## Data Availability

Data are available from the corresponding author on reasonable request.
